# Epidemic trends of dyslipidemia in young adults: a real-world study including more than 20,000 samples

**DOI:** 10.1186/s12944-023-01876-2

**Published:** 2023-07-29

**Authors:** Liang-Yu Liu, Xiyidan Aimaiti, Ying-Ying Zheng, Xiao-Yu Zhi, Zhi-Long Wang, Xin Yin, Ying Pan, Ting-Ting Wu, Xiang Xie

**Affiliations:** 1 Xinjiang Medical University, 830000 Urumqi, China; 2 Department of Cardiology, First Affiliated Hospital of Xinjiang Medical University, 830054 Urumqi, China

**Keywords:** Dyslipidemia, Young, Age, Obesity, Trend

## Abstract

**Background:**

There is an urgent need to learn more about the epidemiological features of dyslipidemia in youth to address the high burden of cardiovascular disease.

**Methods:**

This experiment was an observational, cross-sectional study. The samples were collected from 22,379 college students at Xinjiang Medical University.

**Result:**

The overall prevalence of dyslipidemia was 13.17%, which was significantly higher in men (23%) than in women (7.2%), *p* < 0.01. Similarly, the prevalence rate of obesity in men (11.4%) was significantly higher than that in women (3.4%). The composition of blood lipids, such as triglyceride (TG), total cholesterol (TC), and low density lipoprotein cholesterol (LDL-C), began to increase gradually from the age of 22 and showed a sharp increase after the age of 30; however, a reverse trend was present in high density lipoprotein cholesterol (HDL-C). In terms of the proportion of dyslipidemia in both men and women, low HDL-C accounted for the largest proportion (74%), followed by elevated TGs (14.5%). The overall distribution of rates of dyslipidemia and excess weight showed a U-shaped trend with increasing age, with the lowest rates seen in the 20–24 age group.

**Conclusion:**

Our study sheds light on the epidemiological features of dyslipidemia in young adults and enriches the limited data available on dyslipidemia, providing a reference for the close monitoring and control of risk factors to reduce the occurrence and progression of atherosclerotic cardiovascular disease events.

## Introduction

Atherosclerotic cardiovascular disease (ASCVD) has become the most intractable health problem in the world [1, 2]. Advanced age appears to be one of the most critical risk factors for ASCVD [3]. However, recent studies have shown an increase in the prevalence of ASCVD in young people [4, 5]. As an established hazard element of ASCVD, dyslipidemia contributes to the formation of atherosclerotic plaques and a prothrombotic state, increasing the risk of adverse events such as cardiovascular events [6–8]. There is substantial evidence that lipid disorders are associated with subclinical atherosclerosis in young adults, suggesting that atherosclerosis develops early in life [9, 10]. Dyslipidemia in youth has been shown to persist or persist into adulthood, with the principal link to an increased risk of cardiovascular disease [11, 12]. As the most efficient prevention strategy is to control the key risk elements of ASCVD early before onset and progression [13], it is particularly crucial to intervene early in dyslipidemia.

Due to the unhealthy living habits of contemporary youth [14], dyslipidemia is widespread throughout the world. In terms of the prevalence of dyslipidemia, a study of 2,283 Chinese children and adolescents showed a prevalence of dyslipidemia of 20.6% [15]. Another study showed that among 1783 children and adolescents from northwest China, the prevalence of one and two types of dyslipidemia was 43.2% and 12.2%, respectively [16]. A recent study assessing the prevalence of dyslipidemia among adolescents in all 13 districts of Saudi Arabia revealed that one in four adolescents in Saudi Arabia suffers from dyslipidemia [17]. According to a large survey of 43,368 Chinese adults aged 18 years or older from 2007 to 2010, the awareness rate of dyslipidemia was 31.0%, indicating a low awareness, treatment and control rate of dyslipidemia among Chinese adults [18]. To reverse the dramatic increase in the prevalence of dyslipidemia, early detection, identification and treatment of youthful individuals at risk of dyslipidemia may significantly reduce the lifetime risk of ASCVD [19]. Although the importance of dyslipidemia in young people has been recognized, there is still a lack of epidemiological studies to further reveal the early changes and epidemic trends of blood lipids. Thus, our study retrospectively collected the physical examination data of 22,379 young adults to reveal the characteristics of blood lipids to provide a reliable theoretical basis for the prevention and control of dyslipidemia and obesity in young people.

## Methods

### Study design and subjects

The experiment was an observational, cross-sectional study in which samples were collected from university students undergoing physical examination at the Physical Examination Center of Xinjiang Medical University, including 22,379 Han, Uygur, and Kazakh students. Exclusion criteria included the following: (1) no blood samples or missing sample data; (2) use of cholesterol-lowering drugs; and (3) underlying medical conditions (including hematological disorders, renal disease, active or chronic inflammation, autoimmune diseases, active infection, recent transfusions, cancer).

A health questionnaire was conducted by trained investigators, including demographic characteristics (name, sex, age, ethnicity) and personal and family history of disease. Height, weight, and blood pressure were measured according to standard methods, and body mass index (BMI) was calculated. Blood samples were taken from subjects who fasted for 8–12 h overnight. Fasting blood glucose (FBG), serum total cholesterol (TC), triglyceride (TG), low-density lipoprotein cholesterol (LDL-C), high-density lipoprotein cholesterol (HDL-C) and other blood biochemical indicators were measured by an automated clinical chemical analyzer in the physical examination center of the First Affiliated Hospital of Xinjiang Medical University.

According to the 2016 Guidelines for the prevention and treatment of dyslipidemia in Chinese adults, the diagnostic criteria for dyslipidemia were as follows [20]. High TC (HTC) was defined as ≥ 6.22 mmol/L, high LDL-C (HLDL-C) as ≥ 4.14 mmol/L, low HDL-C (LHDL-C) as ≤ 1.04 mmol/L, and high TG (HTG) as ≥ 2.26 mmol/L. According to the recommendations of the Working Group on Obesity in China, individuals were divided into 3 groups: 18.5 kg/m^2^ ≤ BMI < 24.0 kg/m^2^ (normal weight), 24.0 kg/m2 ≤ BMI < 28.0 kg/m^2^ (overweight), and BMI ≥ 28.0 kg/m^2^ (obese) [21]. Students under 18 years old are grouped according to the criteria of 17 years old according to “Screening for overweight and obesity among school-age children and adolescents” (WS/T 586–2018).

### Statistical analyses

Data were analyzed using SPSS 22 software. Kolmogorov‒Smirnov tests were used to evaluate the normality of continuous variables. To compare groups based on measured data with a normal distribution, the independent sample t test was employed. The enumeration data were expressed as percentages, and the chi-square test was used to compare groups. We compared parametric patient characteristics using one-way ANOVA and nonparametric characteristics using Kruskal‒Wallis analysis. *P* < 0.05 was considered statistically significant.

## Results

### Basic situation

**Fig. 1:**
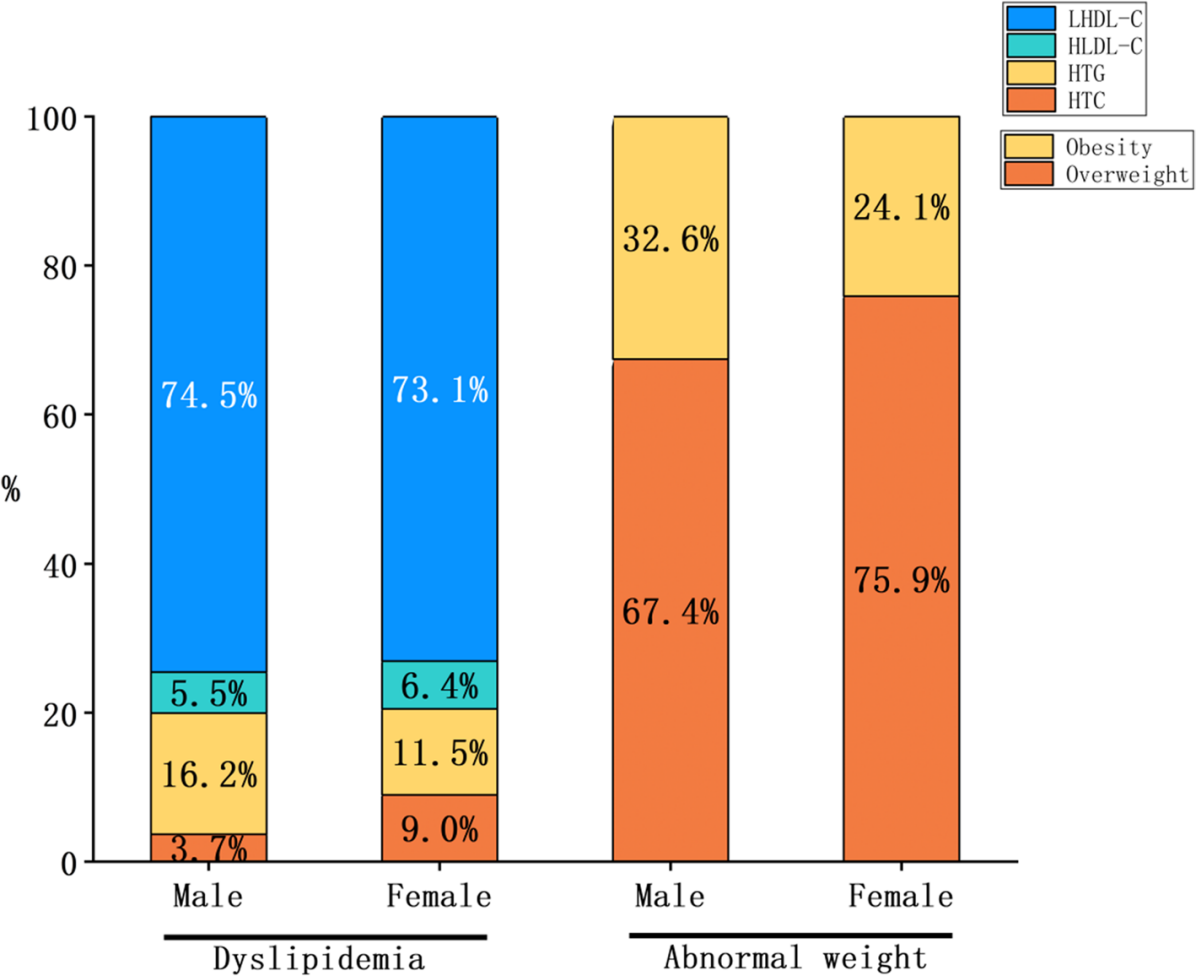
The proportion of male and female with different abnormal lipid components and the proportion of obese and overweight

**Fig. 2:**
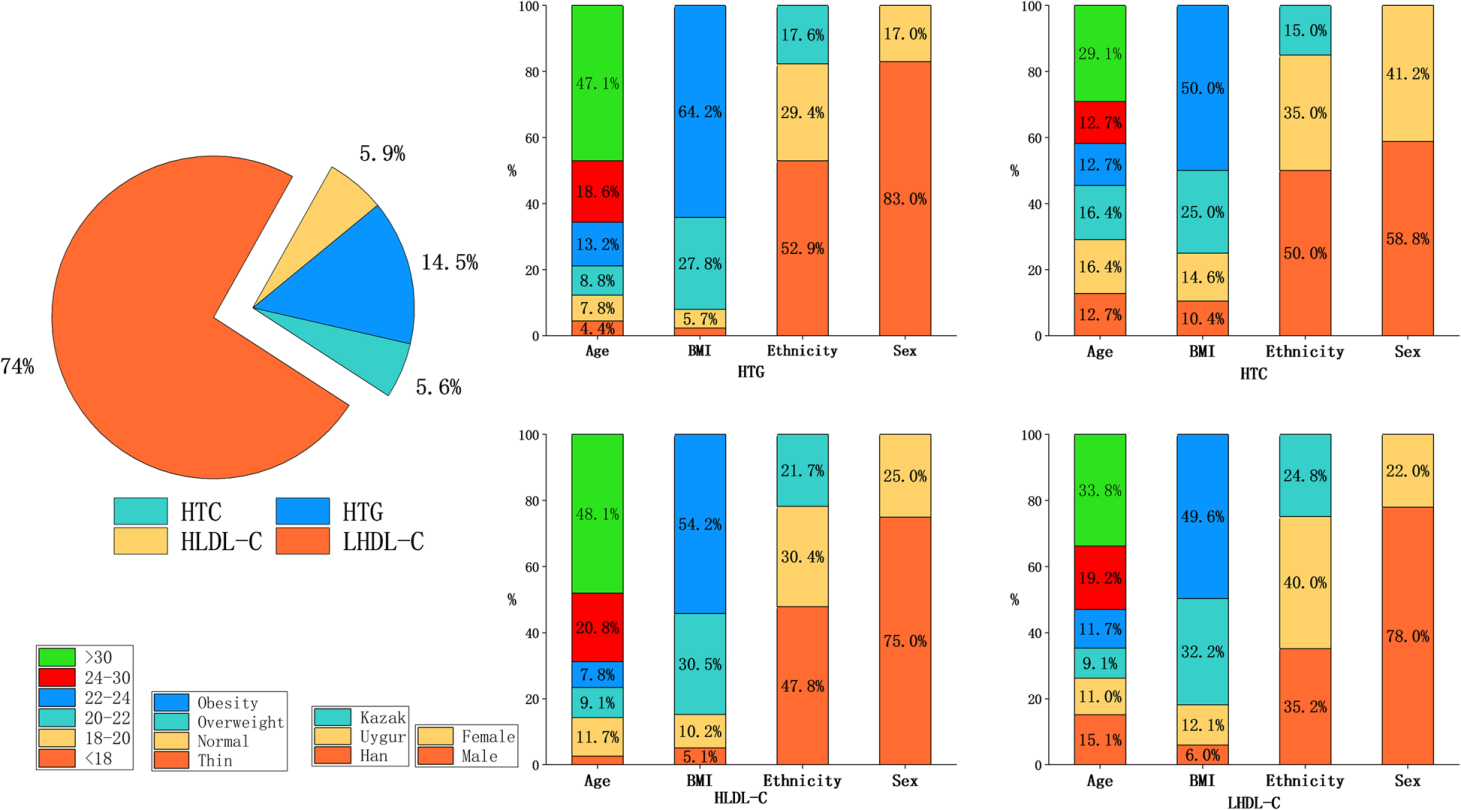
The incidence ratio of different abnormal lipid components in different age, nutritional status, ethnicity and gender groups

Table 1 lists the baseline characteristics and mean values of the lipid profiles of the enrolled population, grouped by sex. The average age of 22,379 subjects was 20.91 ± 2.55 years old, including 8438 males (37.7%). Compared with women, men had higher mean TG and LDL-C and lower mean HDL-C. The average BMI level of males was markedly higher than that of females, and the overall proportion of obesity was also higher in men (11.4%) than in women (3.4%). The average FBG and mean values of systolic and diastolic blood pressure were also higher in men than in women (*P* < 0.001, Table 1). The distribution of the proportion of dyslipidemia and excessive weight grouped by sex is shown in Fig. 1. Then, we subdivided dyslipidemia into the following four categories, namely, HTG, HTC, HLDL-C, and LHDL-C, according to the major lipid component. We can see that the number of people suffering from LHDL-C was the largest, and low HDL-C seems to be the critical composition abnormality accounting for dyslipidemia. Regardless of the kind of lipid component abnormality, the proportion of males, elderly individuals, and obese individuals was relatively higher. More details of the constitutive characteristics of each lipid component abnormality are shown in Fig. 2.

**Table 1:** Baseline characteristics of study subjects and gender subgroups

Variables	Total (*n* = 22,379)	Male (*n* = 8438)	Female (*n* = 13,941)	*P*
**Age,years**	20.91 ± 2.55	20.81 ± 0.70	20.97 ± 2.44	*P* < 0.001
**BMI,kg/m** ^**2**^				
mean ± SD	21.81 ± 3.59	23.12 ± 3.96	21.02 ± 3.09	*P* < 0.001
Thin	3331(14.9%)	759(9.0%)	2572(18.4%)	*P* < 0.001
Normal weight	14,137(63.2%)	4724(56.0%)	9413(67.5%)	
Overweight	3480(15.6%)	1992(23.6%)	1488(10.7%)	
Obesity	1431(6.4%)	963(11.4%)	468(3.4%)	
**SBP,mmHg**				
mean ± SD	113 ± 13	120 ± 12	110 ± 11	*P* < 0.001
< 140	22,047(98.5%)	8161(96.7%)	13,886(99.6%)	*P* < 0.001
≥ 140	332(1.5%)	277(3.3%)	55(0.4%)	
**DBP,mmHg**				
mean ± SD	71 ± 9	74 ± 9	70 ± 8	*P* < 0.001
< 90	21,682(96.9%)	7923(93.9%)	13,759(98.7%)	*P* < 0.001
≥ 90	697(3.1%)	515(6.1%)	182(1.3%)	
**FBG,mmol/L**				
mean ± SD	4.52 ± 0.54	4.53 ± 0.60	4.52 ± 0.49	*P* < 0.001
< 5.60	21,959(98.1%)	8221(97.4%)	13,738(98.5%)	*P* < 0.001
5.6–6.99	384(1.7%)	198(2.3%)	186(1.3%)	
≥ 7.00	36(0.2%)	19(0.2%)	17(0.1%)	
**TC,mmol/L**				
mean ± SD	3.95 ± 0.96	3.95 ± 0.81	3.96 ± 0.73	*P* < 0.001
< 6.22	22,191(99.2%)	8352(99.0%)	13,839(99.3%)	*P* = 0.023
≥ 6.22	188(0.8%)	86(1.0%)	102(0.7%)	
**TG,mmol/L**				
mean ± SD	0.92 ± 0.53	1.08 ± 0.66	0.83 ± 0.40	*P* < 0.001
< 2.26	21,888(97.8%)	8067(95.6%)	13,821(99.1%)	*P* < 0.001
≥ 2.26	491(2.2%)	371(4.4%)	120(0.9%)	
**LDL-C,mmol/L**				
mean ± SD	2.38 ± 0.63	2.49 ± 0.68	2.32 ± 0.59	*P* < 0.001
< 4.14	22,179(99.1%)	8313(98.5%)	13,866(99.5%)	*P* < 0.001
≥ 4.14	200(0.9%)	125(1.5%)	75(0.5%)	
**HDL-C,mmol/L**				
mean ± SD	1.39 ± 0.30	1.27 ± 0.27	1.47 ± 0.30	*P* < 0.001
> 1.04	19,882(88.8%)	6735(79.8%)	13,147(94.3%)	*P* < 0.001
≤ 1.04	2497(11.2%)	1703(20.2%)	794(5.7%)	
**Dyslipidemia**	2947(13.17%)	1944(23.0%)	1003(7.2%)	*P* < 0.001
**ALT,U**/L	17.12 ± 15.65	22.69 ± 20.05	13.74 ± 10.93	*P* < 0.001
**AST,U**/L	18.95 ± 7.55	20.86 ± 9.33	17.79 ± 5.94	*P* < 0.001
**TBIL,umol/L**	13.35 ± 7.05	15.82 ± 7.94	11.85 ± 5.98	*P* < 0.001
**CR,mmol/L**	69.39 ± 13.94	81.51 ± 11.49	62.05 ± 9.45	*P* < 0.001
**BUN,mmol/L**	4.25 ± 1.16	4.68 ± 0.60	3.99 ± 1.07	*P* < 0.001
**HB,g/L**	142 ± 18	159 ± 10	132 ± 13	*P* < 0.001
**WBC,×10** ^**9**^ **/L**	6.70 ± 1.58	6.79 ± 1.56	6.65 ± 1.60	*P* = 0.010
**PLT,×10** ^**9**^ **/L**	274 ± 62	259 ± 55	284 ± 65	*P* < 0.001

*BUN *Blood urea nitrogen, *CR *Creatinine, *UA *Uric acid, *FBG *Fasting blood glucose, *TG *Triglyceride, *TC *Total cholesterol, *HDL-C *High-density lipoprotein cholesterol, *LDL-C *Low-density lipoprotein cholesterol, *SBP *Systolic blood pressure, *DBP *Diastolic blood pressure, *TBIL *Total bilirubin, *HB *Hemoglobin, *WBC *White blood cell, *PLT *Platelet.

### Grouping by age group

**Fig. 3:**
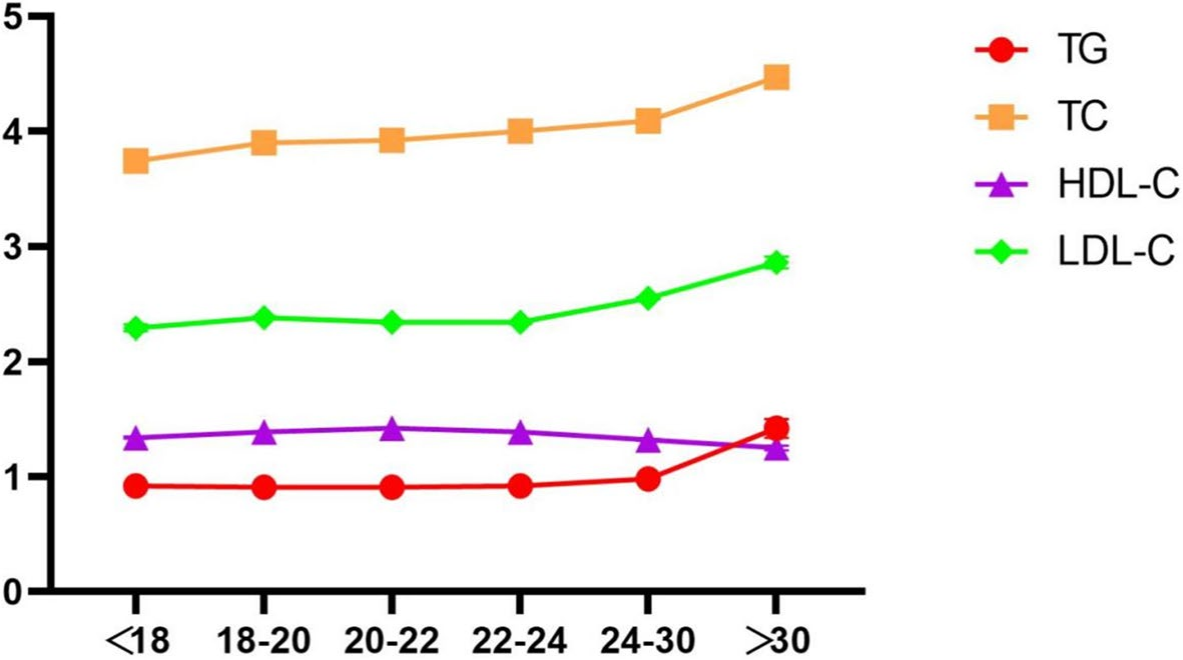
Trend of different lipid components with age

**Fig. 4:**
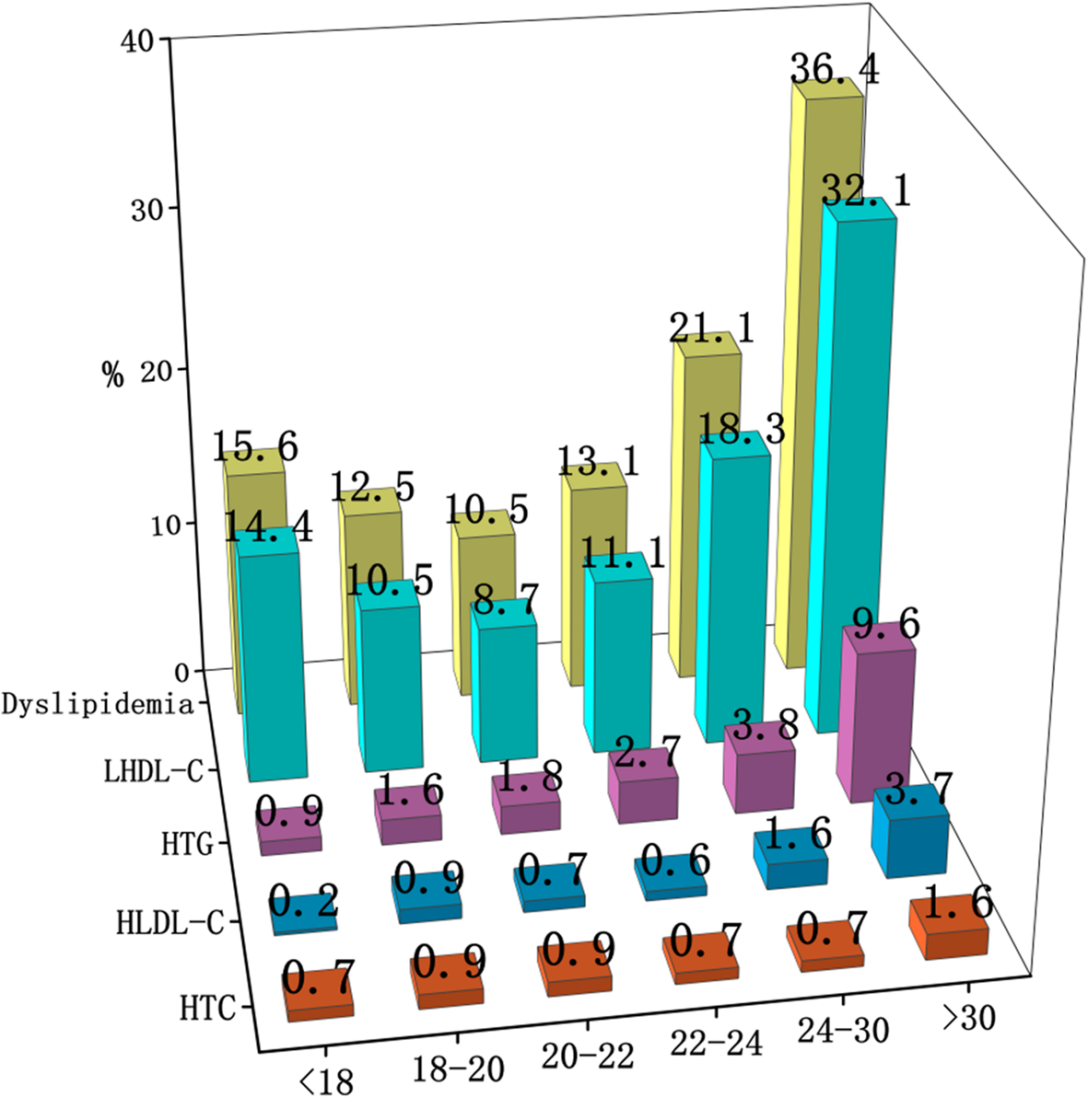
Prevalence of dyslipidemia in different age groups

**Fig. 5:**
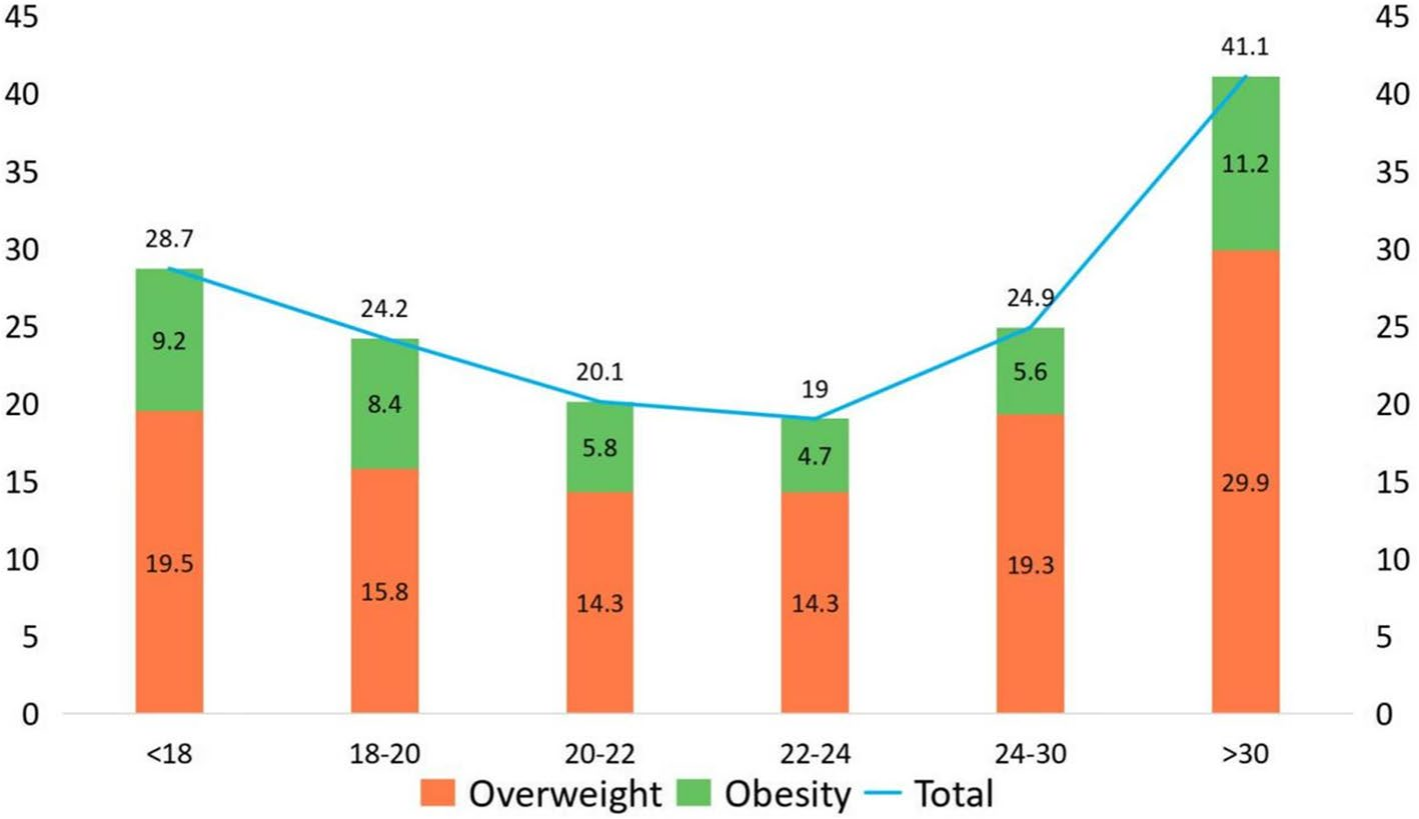
Prevalence of obesity and overweight in different age groups

With increasing age, the average levels of TG, TC and LDL-C tended to gradually increase and showed a sharp increase when age > 30, while HDL-C showed the opposite trend (Fig. 3). The prevalence of dyslipidemia was highest in the age group > 30 (36.4%), followed by the age group 24–30 (21.1%), and the lowest prevalence was in the age group 20–22 (10.5%). Simultaneously, the group over 30 years old had the highest concentration of overweight and obese students (41.1%) and higher ratios of abnormal FBG, blood pressure and lipid components (Table 2). The overall distribution of the prevalence of dyslipidemia in all age groups showed a “U-shaped curve”, so as LHDL-C. (See Fig. 4). Overweight and obese students were mostly < 18 years old and > 30 years old, with the largest proportion being > 30 years old (41.1%) and the smallest proportion being 22–24 years old (19.0%), and the overall distribution was close to a U-shaped trend (Fig. 5).

**Table 2:** Prevalence of dyslipidemia by age group

Variables	< 18 (*n* = 436)	18–20 (*n* = 6686)	20–22 (*n* = 7889)	22–24 (*n* = 4543)	24–30 (*n* = 2638)	> 30 (*n* = 187)	*P*
**BMI**							
Thin	73(16.7%)	1141(17.1.%)	1199(15.2%)	616(13.6%)	295(11.2%)	7(3.7%)	*P* < 0.001
Normal weight	238(54.6%)	3928(58.7%)	5107(64.7%)	3065(67.5%)	1688(64.0%)	103(55.1%)	
Overweight	85(19.5%)	1057(15.8%)	1128(14.3%)	648(14.3%)	508(19.3%)	56(29.9%)	
Obesity	40(9.2%)	560(8.4%)	455(5.8%)	214(4.7%)	147(5.6%)	21(11.2%)	
**FBG**							
< 5.60	430(98.6%)	6589(98.5%)	7714(97.8%)	4451(98.0%)	2601(98.6%)	174(93.0%)	*P* < 0.001
5.6–6.99	3(0.7%)	86(1.3%)	164(2.1%)	86(1.9%)	36(1.4%)	9(4.8%)	
≥ 7.00	3(0.7%)	11(0.2%)	11(0.1%)	6(0.1%)	1(0.0%)	4(2.1%)	
**HTC**	3(0.7%)	58(0.9%)	72(0.9%)	34(0.7%)	18(0.7%)	3(1.6%)	*P* = 0.663
**HTG**	4(0.9%)	106(1.6%)	141(1.8%)	121(2.7%)	101(3.8%)	18(9.6%)	*P* < 0.001
**HLDL**	1(0.2%)	62(0.9%)	58(0.7%)	29(0.6%)	43(1.6%)	7(3.7%)	*P* < 0.001
**LHDL**	63(14.4%)	699(10.5%)	686(8.7%)	506(11.1%)	483(18.3%)	60(32.1%)	*P* < 0.001
**Dyslipidemia**	68(15.6%)	834(12.5%)	827(10.5%)	594(13.1%)	556(21.1%)	68(36.4%)	*P* < 0.001
**SBP ≥ 140mmHg**	9(2.1%)	103(1.5%)	137(1.7%)	60(1.3%)	18(0.7%)	5(2.7%)	*P* = 0.002
**DBP ≥ 90mmHg**	7(1.6%)	160(2.4%)	272(3.4%)	172(3.8%)	70(2.7%)	16(8.6%)	*P* < 0.001

### Grouping by BMI or different ethnic groups

Based on BMI, we divided the students into four groups. The prevalence rate of dyslipidemia increased with increasing BMI. In comparison, we found that rates of dyslipidemia, abnormalities in individual lipid components, and elevation of blood pressure and blood glucose were more common in the obese and overweight groups (Table 3).

**Table 3:** Prevalence of dyslipidemia according to nutritional status by BMI

Variables	Thin (*n* = 3331)	Normal (*n* = 14,129)	Overweight (*n* = 3482)	Obesity (*n* = 1437)	*P*
**HTC**	18(0.5%)	92(0.7%)	43(1.2%)	35(2.4%)	*P* < 0.001
**HTG**	13(0.4%)	146(1.0%)	169(4.9%)	163(11.3%)	*P* < 0.001
**HLDL-C**	9(0.3%)	82(0.6%)	63(1.8%)	46(3.2%)	*P* < 0.001
**LHDL-C**	131(4.0%)	1141(8.1%)	749(21.5%)	476(33.1%)	*P* < 0.001
**Dyslipidemia**	164(4.9%)	1352(9.6%)	868(24.9%)	563(39.2%)	*P* < 0.001
**SBP**					
< 140mmHg	3310(99.4%)	14,010(99.2%)	3388(97.3%)	1339(93.2%)	*P* < 0.001
≥ 140mmHg	21(0.6%)	119(0.8%)	94(2.7%)	98(6.8%)	
**DBP**					
< 90mmHg	3285(98.6%)	13,841(98.0%)	3299(94.7%)	1252(87.5%)	*P* < 0.001
≥ 90mmHg	46(1.4%)	288(2.0%)	183(5.3%)	180(12.5%)	
**FBG**					
< 5.60	3276(98.3%)	13,916(98.5%)	3401(97.7%)	1366(95.0%)	*P* < 0.001
5.6–6.99	54(1.6%)	196(1.4%)	74(2.1%)	60(4.2%)	
≥ 7.00	1(0.0%)	17(0.1%)	7(0.2%)	11(0.8%)	

Among the three ethnic groups, the prevalence of dyslipidemia was highest in Uyghur students and lowest in Kazak students (*P* < 0.001, Table 4). Except for the highest prevalence of LHDL-C in Uygur students, Han students were more vulnerable to HTC, HTG and HLDL-C and were more likely to suffer from overweight or obesity and higher blood pressure.

**Table 4:** Prevalence of dyslipidemia in different ethnic groups

Variables	Han (*n* = 13,327)	Uygur (*n* = 7515)	Kazak (*n* = 1536)	*P*
**BMI**				
Thin	1918(14.4%)	1204(16.0%)	209(13.6%)	*P* < 0.001
Normal weight	8093(60.7%)	4986(66.3%)	1058(68.9%)	
Overweight	2268(17.0%)	1013(13.5%)	198(12.9%)	
Obesity	1048(7.9%)	312(4.2%)	71(4.6%)	
**FBG**				
< 5.60	13,052(97.9%)	7394(98.4%)	1512(98.4%)	*P* = 0.163
5.6–6.99	250(1.9%)	112(1.5%)	22(1.4%)	
≥ 7.00	25(0.2%)	22(0.1%)	2(0.1%)	
**HTG**	365(2.7%)	112(1.5%)	14(0.9%)	*P* < 0.001
**HTC**	131(1.0%)	53(0.7%)	4(0.3%)	*P* = 0.004
**HLDL-C**	142(1.1%)	50(0.7%)	8(0.5%)	*P* = 0.004
**LHDL-C**	1446(10.9%)	932(12.4%)	119(7.7%)	*P* < 0.001
**Dyslipidemia**	1773(13.3%)	1038(13.8%)	136(8.9%)	*P* < 0.001
**SBP ≥ 140mmHg**	248(1.9%)	59(0.8%)	25(1.6%)	*P* < 0.001
**DBP ≥ 90mmHg**	500(3.8%)	148(2.0%)	49(3.2%)	*P* < 0.001

## Discussion

In this study, we reveal several characteristic findings regarding lipids by analyzing a relatively large sample size of adolescent college students. First, we confirmed that the overall prevalence of dyslipidemia is as high as 13.17% and obviously higher in males (23%) than in females (7.2%). Second, we found that low HDL-C was the main type of dyslipidemia in youthful people (proportion of attribution up to 74%). In addition, we have some extended findings. Our results indicate that the prevalence of dyslipidemia, including the prevalence of low HDL-C, and the proportion of obese or overweight individuals show a U-shaped trend with increasing age. The prevalence of dyslipidemia starts to increase gradually from the age of 22 and shows a sharp increase after 30 years. Based on our results, we call for early attention to dyslipidemia.

Previous research on dyslipidemia has focused on the middle-aged and older age groups, but several studies in recent years have pointed to the development of dyslipidemia that dates back to adolescence [22]. Liu et al. reported a prevalence of dyslipidemia of 29.09% in 18- to 40-year-olds [23]. Our subjects were much younger, and the prevalence was as high as 13.17%. Consistent with previous literature [24, 25], we found significantly higher rates of dyslipidemia in males because males may have additional risk factors, such as smoking, alcohol consumption, and hypertension. Hormones may be another underlying reason, as reports have shown that estrogen significantly inhibits low-density lipoprotein transport by downregulating endothelial scavenger receptor B1 (SR-B1) [26, 27]. We then found that among the types of dyslipidemia in young adults, the most significant was low HDL-C, which was up to 9.99%, which consisted of the previously reported findings of Zhang et al. [28]. The underlying mechanism of this, possibly unhealthy lifestyle habits such as diet, smoking and drinking, can have a huge impact on blood triglycerides, cholesterol, and LDL-C. However, the effect on HDL-C is smaller. HDL-C levels decline almost linearly throughout adolescence, which may be closely related to physiological changes in the levels of sex hormones during adolescence [29]. By comparing the prevalence of dyslipidemia among students of different ethnic groups, we conclude that the prevalence of dyslipidemia is higher among Uyghur students than in the other two groups, which may be caused by differences in genetic factors, lifestyle factors, and dietary habits [30]. We also found that obese or overweight students had higher rates of dyslipidemia. A possible explanation is that obese people have a higher rate of adipose breakdown in their visceral fat cells than normal people, producing more free fatty acids, which enter the liver through the portal vein system and increase TG synthesis. In addition, in terms of social roles, college students do not have the extra energy and time for moderate exercise due to intense study pressure. The availability of a wide variety of fried foods, barbecued foods and high-calorie drinks favored by college students has made it easy for students to consume on a long-term, large-scale basis, leading to obesity. Consequently, efforts to control risk factors in the adolescent population, promoting reasonable exercise and a healthy diet are the key to preventing dyslipidemia [31].

By performing a population analysis of blood lipids in young people of different ages, it was found that the composition of various lipids begins to change significantly from around the age of 22, with increasing age. The proportion of overweight and obese people varies as a U-shaped curve with age, which essentially coincides with the abovementioned trend in the prevalence of dyslipidemia. This suggests that obesity is also an essential factor in the development of dyslipidemia in young individuals [32, 33]. It is therefore essential for young people at this age to return to normal dietary habits, eat a balanced diet, establish an active lifestyle and engage in appropriate physical exercise to reduce the prevalence of dyslipidemia.

## Study strengths and limitations

Our study reveals the epidemiological features of dyslipidemia in western China and enriches the data of prevalence of dyslipidemia in young adults. The large sample sizes of more than 20 000 cases strength the credibility of our study. Nonetheless, there are some limitations to this study. First, these conclusions are based on a cross-sectional analysis, so it is not possible to accurately characterize the causal relationship between the relevant factors and dyslipidemia. Second, the study was conducted on physical examination data from a single center, and the results were limited to the entire university population. Third, additional data are currently not available on confounding factors that may affect lipid levels, including hormone levels, dietary characteristics, levels of physical activity, and other lifestyle habits, and further studies are needed to clarify whether different lifestyles produce clinically significant changes in lipid concentrations. Despite its limitations, the strength of this study lies in the inclusion of health examination data from a large number of healthy college students and in the stringent requirements placed on the data collection personnel to ensure that high-quality data were collected.

## Conclusion

Collectively, this study demonstrates a relatively high prevalence of dyslipidemia among adolescent students. Dyslipidemia has its own characteristics among different ethnic groups, different genders, and different BMI groups. Our study enriches the limited data available on dyslipidemia and highlights the need for early prevention and treatment in adolescents.

## Data Availability

Due to confidentiality policies, data will not be shared.
